# The Negative Effect of Job Insecurity in the Virtuous Cycle Between Trust in the Organization, Subjective Well-Being, and Task Performance in the Current Volatility, Uncertainty, Complexity, and Ambiguity Context

**DOI:** 10.3389/fpsyg.2021.796669

**Published:** 2021-12-22

**Authors:** Živilė Stankevičiūtė, M. Isabel Sanchez-Hernandez, Eglė Staniškienė

**Affiliations:** ^1^Sustainable Management Research Group, School of Economics and Business, Kaunas University of Technology, Kaunas, Lithuania; ^2^School of Economics and Business Administration, University of Extremadura, Badajoz, Spain

**Keywords:** job insecurity, trust in the organization, subjective well-being, employee performance, task performance, VUCA

## Abstract

Over the past decade, job insecurity referring to the employees’ perceived threat to the continuity and stability of employment as it is currently experienced has become a hot topic. A general assumption, supported by the findings, is that job insecurity causes far-reaching negative consequences for the employee health and well-being, attitudes toward organization and the job, and behaviors at work. However, the focus on behavioral outcomes, especially on employee performance at work, is still scant. Moreover, the literature remains fragmented concerning the impact of job insecurity on employee trust in the organization and how the trust influences employee subjective well-being (SWB), which in turn affects employee performance. Consequently, the link between job insecurity and SWB needs more investigation. Trying to narrow the gap, the paper aims at revealing the linkage between job insecurity, trust in the organization, SWB, and task performance. Quantitative data were collected in Lithuania. As predicted, the results revealed that job insecurity had a negative impact on trust in the organization and employee SWB. In case of linkage between job insecurity and task performance, the hypothesis was rejected. In general, these findings affirmed that job insecurity was a hindrance stressor, which needed to be considered when managing human resources in the current volatility, uncertainty, complexity, and ambiguity context.

## Introduction

For a couple of centuries, work has become a subject of transformations (Sverke and Hellgren, 2002), especially recently referring to volatility, uncertainty, complexity, and ambiguity (VUCA), context (Baran and Woznyj, 2020). Rapid technological advancement coupled with the general ambition within organizations to save costs and increase effectiveness (Flecker et al., 2017; Lee et al., 2018; Sverke et al., 2019) causes employees’ feelings of insecurity about the nature and future existence of their job (Sverke and Hellgren, 2002). In contemporary organizational settings and societies, job insecurity, in terms of quantitative job insecurity (threats to the continuation or loss of the job itself), and in terms of qualitative job insecurity (threats to the continued existence of valued aspects of the job; De Witte, 2005), is considered as an important job stressor, leading to significant negative consequences for employees (Vander Elst et al., 2014a; Lee et al., 2018). While the previous studies have provided evidence of detrimental effects of job insecurity on work-related attitudes and health and well-being outcomes, and behavioral outcomes (Sverke et al., 2019), some gaps remain nonetheless.

First, assuming the existence and the relevance of the two types of job insecurity, quantitative job insecurity still receives considerable attention compared to qualitative (Vander Elst et al., 2014b). Trying to narrow the gap and treating job insecurity as a complex phenomenon, the current paper treats job insecurity as a second-order construct, which consist of both types of insecurity.

Second, referring to outcomes of job insecurity, the paper responds to the previous calls in the literature to narrow the gap and to investigate how job insecurity is related to trust in the organization (Kim, 2019), subjective well-being (SWB; Hu et al., 2021), and task performance (Sverke et al., 2019). Given that trust in the organization is at the heart of employment relations (Guest, 2004), task performance encompasses the quantity and the quality of work (Sverke et al., 2019), and the SWB reflects the person’s feelings about life as measured by their own standards (Diener and Ryan, 2009), the relationship between job insecurity and the mentioned outcomes becomes highly relevant in the VUCA world (Millar et al., 2018).

Finally, empirical evidence regarding the linkage between trust in the organization and SWB (Oliveira et al., 2020) and between SWB and task performance (Peiró et al., 2019) is scant. They do not provide an explicit message about the nature of the relationship and due to this require further investigation.

Considering the gaps illustrated above, the aim of the paper is to reveal the linkage between job insecurity, trust in the organization, SWB, and task performance in the VUCA context. In doing this, the paper seeks to answer the following: (a) Will job insecurity impact trust in the organization, SWB, and task performance? (b) Will trust in the organization impact SWB and accordingly will SWB impact task performance? In order to answer these questions, this paper analyzes data of employees’ perceptions from a survey carried out in Lithuania.

The current paper is supposed to make three main contributions to the existing body of knowledge. First, the paper intends to enrich the job insecurity literature by identifying how it affects employee behavior in terms of task performance, work-related attitudes in terms of trust in the organization, and well-being in terms of SWB. Second, given that the previous literature differentiates quantitative and qualitative job insecurity (De Witte, 2005), the intention is to provide the support for the idea that the job insecurity construct has two dimensions. Third, the paper deals with the virtuous cycle between trust in the organization, SWB, and task performance and captures the expected negative effect caused by job insecurity in it. As such, the complexity of relationships between job insecurity and its outcomes is revealed.

The remainder of the paper is structured as follows. The theoretical part describes four constructs, namely job insecurity, trust in an organization, SWB, and task performance. Later, the hypotheses are developed. Then, the research method applied is described. The empirical results and discussion come next. Finally, conclusions are drawn.

## Theoretical Background and Hypotheses

### Job Insecurity

As a result of the changing nature of the relationship between employee and organization (Piccoli and De Witte, 2015), job insecurity has become a “sizeable social phenomenon” (De Witte, 2005) referring to employees feeling that their jobs are at risk (Reisel, 2003; Vander Elst et al., 2014a; Probst et al., 2017, 2019). In our VUCA times, and more than never in the context of the COVID-19 pandemic, job insecurity presents a management challenge (Murugan et al., 2020).

Job insecurity has been defined in various ways in the literature. One of the earliest and most-quoted definitions was provided by Greenhalgh and Rosenblatt (1984, p. 438), claiming that job insecurity was “the perceived powerlessness to maintain desired continuity in a threatened job situation.” Another commonly quoted definition was proposed by De Witte (2005, p. 1), arguing that job insecurity was “the perceived threat of job loss and the worries related to that threat.” Nonetheless, the diversity in definitions—for the overview of definitions, see Shoss (2017)—allows to point out several characteristics of the construct included in all or some definitions. First, job insecurity is a subjective experience, resulting from an individual’s perception and interpretation of the actual work situation implying that the same objective situation may be interpreted in various ways by different employees (De Witte et al., 2015). Thus, the following situation is possible: Some employees may feel secure about their jobs, even though they will be laid off soon afterward, whereas others may feel insecure although their job continuity is (“objectively speaking”) not in danger (De Witte, 2005). Second, job insecurity is a future-focused phenomenon (Vander Elst et al., 2014a). Job insecurity reflects a forecast about a loss event, which might happen 1 day in the nearest or further future (Shoss, 2017). Thus, employees are “groping in the dark” as far as their future within the particular organization is concerned (De Witte, 2005). Third, job insecurity hints at involuntary nature (Sverke and Hellgren, 2002) as the construct reflects “discrepancy between what people wish for (certainty about the future of their current employment) and what people ‘get’ (the perception that the current job is threatened)” (De Witte et al., 2015, p. 110). Fourth, job insecurity implies uncertainty about the future: The employee does not know whether they will keep or lose the current job (De Witte et al., 2015). Finally, a feeling of powerlessness is also a part of numerous job insecurity definitions (De Witte, 2005).

The overview of definitions (Shoss, 2017) also allows for distinguishing different types of job insecurity, namely quantitative and qualitative. Quantitative job insecurity denotes the fear of losing the job as such (Vander Elst et al., 2014a). Employees are uncertain about whether they will be able to keep their current jobs or will become unemployed (De Witte, 2005). Thus, quantitative job insecurity implies the worries about losing one’s job altogether (Hu et al., 2021). Meanwhile, qualitative job insecurity refers to employees’ perceived threat to valued job features (Vander Elst et al., 2014a). Thus, employees are not so much afraid of being fired, but rather fear the impairment of valued job features, such as career possibilities, development of competencies, or salary (Hu et al., 2021).

### Task Performance

Being a central construct in Industrial/Organisational Psychology, employee performance refers to “actions, behaviour and outcomes that employees engage in or bring out that are linked with and contribute to organizational goals” (Viswesvaran and Ones, 2000, p. 216). In other words, employee performance defines whether the behavior of employees matches the goals of the particular organization and whether it can achieve the desired results of that organization (Gong et al., 2019). Actually, employee performance is an umbrella term, which includes several distinct types or dimensions of performance behavior (Sverke et al., 2019). This paper limits its focus only to one dimension, namely task performance.

In Work Psychology literature, task performance is defined as the effectiveness with which job incumbents perform activities that contribute to the organization’s technical core either directly by implementing a part of its technological process, or indirectly by providing it with the necessary materials or services (Borman and Motowidlo, 1993, 1997). Similarly, Van Scotter (2000, pp. 80–81) argues that employees are engaging in task performance when they “use technical skills and knowledge to produce goods or services through the organization’s core technical processes, or when they accomplish specialized tasks that support these core functions.” In general terms, task performance refers to the execution of the tasks assigned to the employee (Darvishmotevali and Ali, 2020) through a job description or communicated in other ways (Sverke et al., 2019). Accordingly, task performance requires more cognitive ability and is primarily facilitated through task knowledge, task skill, and task habits (Conway, 1999). In order to be proficient at task performance and to meet the expectations the organization (Darvishmotevali and Ali, 2020), employees need both, the ability to do the job and prior experience (Pradhan and Jena, 2017).

### Subjective Well-Being

Well-being, understood as the essential qualities of a good society and the good life, has been a subject of consideration at least since the times of Aristotle (Diener and Suh, 1997). Despite alternative viewpoints in determining the quality of life, two conceptual approaches to well-being research now prevail in the field (Western and Tomaszewski, 2016), namely the objective and the subjective approaches. As the objective well-being is based on observable factors such as richness, tangible goods, or health (D’Acci, 2011), the SWB refers to people’s own evaluations of their lives (Western and Tomaszewski, 2016) and is psychologically experienced (D’Acci, 2011). The current paper limits its focus only to SWB.

According to Diener et al. (2002, p. 63), SWB is defined as “a person’s cognitive and affective evaluations of his or her life.” Proctor (2014, p. 6437) claims that SWB “is the personal perception and experience of positive and negative emotional responses and global and (domain) specific cognitive evaluations of satisfaction with life.” Actually, SWB is a more scientific-sounding term for what people usually mean by happiness (Diener et al., 2002; Seligman and Csikszentmihalyi, 2014). SWB is a self-reported measure of well-being and addresses the person’s feelings about life in the context of their own standards (Diener and Suh, 1997; Diener and Ryan, 2009). Accordingly, the evaluations can be either formulated in terms of cognitive reflections or in terms of affect (Diener and Suh, 1997). The cognitive aspect of SWB refers to what people think about their life satisfaction in general (life as a whole) and also in a certain area of life, such as work or relationships. Meanwhile, the affective aspect of SWB implies the individual’s feeling, emotion, and mood. The affect can be positive when things seem to be going well or negative when people experience a decline in the course of things (Diener et al., 2017). Positive affect encompasses both momentary emotions (for instance, enjoyment), and more chronic long-term moods (for instance contentment). In the case of negative affect, the situation is similar, with negative affect including anger, sadness, worry, or stress as momentary emotions, and longer-lasting moods such as depression that might occur over time (Diener et al., 2017).

### Trust in the Organization

Following the notion that trust is a key “aspect of relationships” (Gullett et al., 2009), trust is usually defined as “a psychological state comprising the intention to accept vulnerability based upon positive expectations of the intentions or behaviour of another” (Rousseau et al., 1998, p. 395). To elaborate this idea, there are two conditions that must exist for trust to arise, namely risk and interdependence. Risk is the perceived probability of loss, as interpreted by the trusting party, while interdependence implies that one party’s interests cannot be fulfilled without reliance on the other party (Rousseau et al., 1998).

Within an organizational setting, trust can be manifested in reference to individuals (for example, trust in one’s supervisor or colleague), specific groups (for example, trust in top-level managers or team), or the organization as a whole (Schoorman et al., 2007). The current paper considers employees’ trust in the employing organization as a whole (Verburg et al., 2018; Guzzo et al., 2021). Accordingly, trust in an organization refers to the individual’s expectation that some organized system will act with predictability and goodwill (Maguire and Phillips, 2008).

### Hypotheses Development

**Figure 1:**
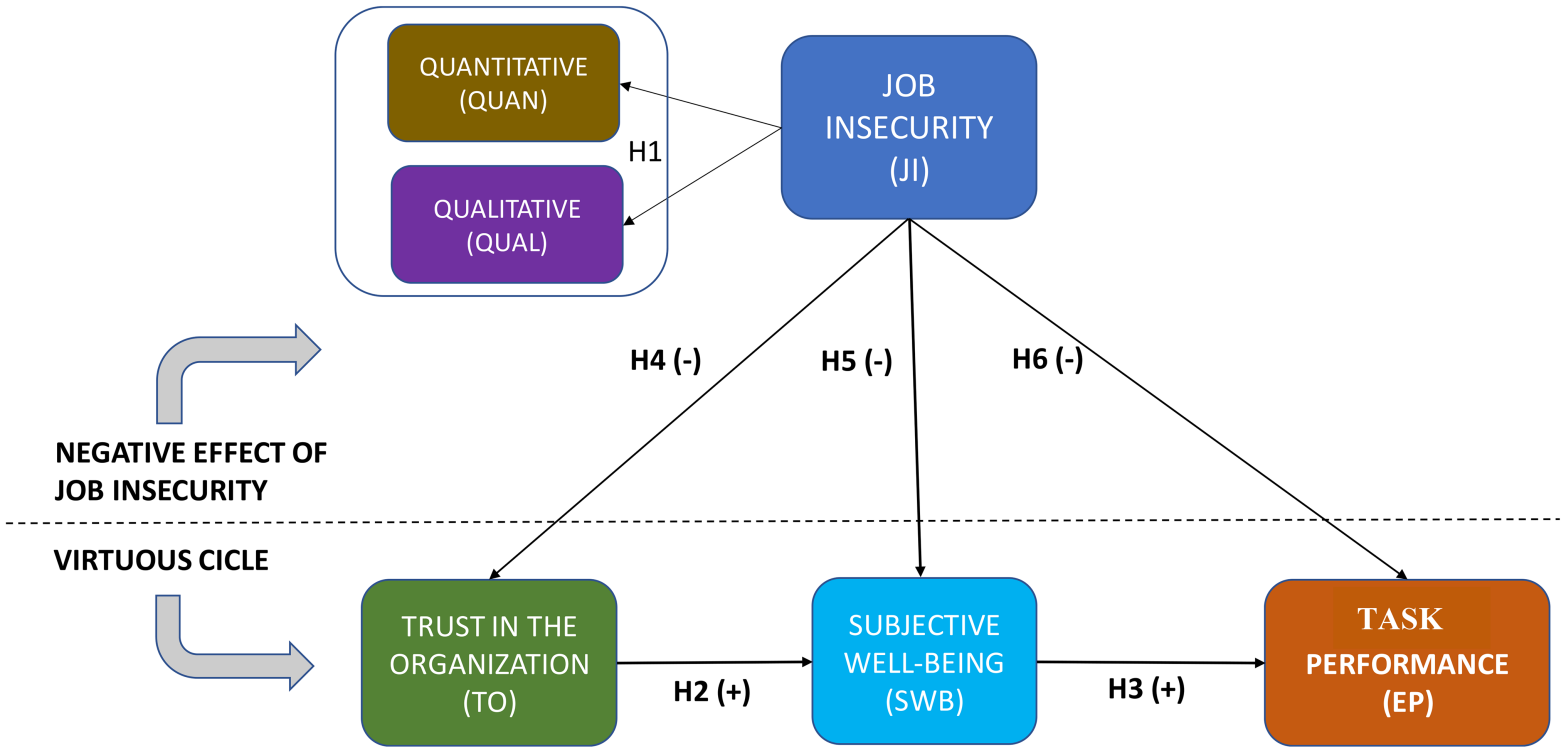
The theoretical model.

The main theoretical model shown in Figure 1 represents, on the one hand, a virtuous cycle between trust in the organization, SWB, and employee performance. On the other hand, the model captures the expected negative effect caused by job insecurity (considering the quantitative and the qualitative dimensions) in this virtuous cycle. Below the hypothesis is grounded.

#### Quantitative and Qualitative Job Insecurity (H1)

The current research follows the view of Shoss (2017, p. 1914), treating job insecurity as “a perceived threat to the continuity and stability of employment as it is currently experienced” while capturing both quantitative and qualitative types of job insecurity. Consequently, the first hypothesis is posited.

**H1:** Job insecurity has two dimensions, one quantitative and other qualitative.

#### Linkage Between Trust in an Organization and Subjective Well-Being (H2)

Trust in the organization implies a healthy employee–employer relationship (Guest, 2004; Richter and Näswall, 2019). When trusting an organization, employees have the confidence that employer will not exploit the employees’ vulnerabilities (Holland et al., 2017). With respect to this, it could be predicted that trust in an organization serves as an antecedent of SWB, referring to people’s cognitive and affective evaluations of their lives. Such prediction is supported by several previous empirical studies. For instance, Oliveira et al. (2020) found that trust in an organization positively correlated with SWB. Based on the above, the current paper hypothesizes the following.

**H2:** Trust in organizations is direct and positively related to SWB

#### Linkage Between SWB and Task Performance (H3)

According to the “happy worker–productive worker” thesis, happy employees perform better than less happy ones (Cropanzano and Wright, 2001). In this sense, the paper argues that employees with high levels of SWB will exhibit higher levels of task performance. Such proposition is supported by some previous empirical findings. For instance, Peiró et al. (2019) found that employees with high levels of happiness were more productive than those with a low level of happiness. Fogaça and Junior (2016) indicated that well-being at work, including positive effect, and job satisfaction were positively associated with individual job performance. The study of Zelenski et al. (2008) provided significant support for the “happy worker-productive worker” thesis demonstrating that happy people achieved a higher level of productivity at both the state and trait levels of analysis. More recently, Lee et al. (2021) revealed that SWB was positively and significantly related to job performance.

Moreover, when exploring the link between SWB and task performance, it is worthwhile to address two notions. As stated by Zelenski et al. (2008, p. 523), “across the various tasks typically required of employees, happiness will, on balance, likely benefit overall productivity.” Additionally, Lee et al. (2021, p. 4) argue that “the positive psychology of SWB gives employees a sense of security, makes them settle down in the job” and accordingly improves task performance.

Consequently, based on theoretical reasoning and prevailing findings from previous studies, the current paper hypothesizes the following:

**H3:** SWB is directly and positively related to task performance

#### Linkage Between Job Insecurity and Trust in an Organization (H4)

Drawing upon the previous literature, it seems that psychological contract theory (Rousseau, 1995) is one of the main theoretical approaches used to explain the relationship between job insecurity and trust in an organization (Richter and Näswall, 2019). Psychological contract refers to the set of explicitly or implicitly given promises including duties and entitlements between the employer and employee, as perceived by the employee (Conway and Briner, 2005). In most countries, including Lithuania, psychological contract is likely to include job security (De Cuyper and De Witte, 2007). In this sense, employees expect that when their endeavors benefit the organization, the organization will in turn reciprocate by offering them rewards in terms of job security (Piccoli and De Witte, 2015). Meanwhile, employees may perceive job insecurity as a breach of the psychological contract (Schreurs et al., 2012). In turn, the breach results in an impairment of the employee–employer relationship, which can manifest as a loss of trust in the organization (Conway et al., 2011).

Supporting this reasoning, various studies found that insecure employees no longer believed that the employer would deliver on its implied obligations and trusted their organizations less (Richter and Näswall, 2019). For instance, the meta-analysis of Cheng and Chan (2008) revealed the negative effect of job insecurity on trust. More recently, Kim (2019) provided findings that job insecurity lowered organizational trust.

Based on the theoretical arguments and research findings presented, the current paper hypothesizes the following.

**H4:** Job insecurity is directly and negatively related to trust in organizations

#### Linkage Between Job Insecurity and SWB (H5)

The current paper employs the conservation of resources (COR) theory (Hobfoll, 1989) to address the relationship between job insecurity and SWB. According to the COR theory, resources are defined as “those objects, personal characteristics, conditions, or energies that are valued by the individual or that serve as a means for attainment of these objects, personal characteristics, conditions, or energies” (Hobfoll, 1989, p. 516). Following this theoretical view, employees strive to retain, gain, and protect their resources (Hu et al., 2021). Meanwhile, job insecurity implies the possibility of losing resources, for instance, lower career possibilities in case of qualitative job insecurity or loss of work position in case of quantitative job insecurity. As such, employees who feel insecure about their jobs will experience lower levels of SWB, because they are faced with the possible loss of important employment-related resources (Hu et al., 2021).

Turning to empirical findings, previous studies have shown that job insecurity correlates with a lower score on various indicators of job-related well-being (Vander Elst et al., 2012; Darvishmotevali and Ali, 2020). More precisely, Stiglbauer and Batinic (2015) pointed out the detrimental effects of job insecurity for an employee’s SWB as job insecurity was associated with lower happiness and higher depression. More recently, similar results were found by Hu et al. (2021), while addressing only qualitative job insecurity, SWB was negatively affected by qualitative job insecurity.

Building upon the theoretical arguments and research results presented, the current paper hypothesizes as follows.

**H5:** Job insecurity is directly and negatively related to SWB

#### Linkage Between Job Insecurity and Task Performance (H6)

As task performance is a key area for managers, gaining a complex understanding of the nature of the relationship between job insecurity and task performance may have far reaching practical implications for organizational sustainability (Piccoli et al., 2021). However, the previous research has demonstrated conflicting findings on the link between the two constructs (Shin et al., 2019; Sverke et al., 2019) as the majority of studies have found job insecurity to be negatively related to general and task performance (Schreurs et al., 2012; Vander Elst et al., 2014a; Roll et al., 2015); this notwithstanding, there are some studies that have found non-significant (Selenko et al., 2017) or even positive associations (Probst et al., 2007). These mixed findings call for the further investigation while referring to the psychological mechanisms, which serve for explaining why job insecurity may lead to particular consequences. In doing this, the current paper relies on the stress theory, more precisely on the hindrance dimension of two-dimensional stressor model (Piccoli et al., 2021). Unquestionably, in contemporary working life, job insecurity is considered as an important stressor (Vander Elst et al., 2014a). Following the mentioned model, any stressor reflects two basic dimensions, hindrance (“bad” stress) and challenge (“good stress”; Lepine et al., 2005). Despite this, the latest empirical studies (for instance, Piccoli et al., 2021) found support only for the negative job insecurity impact on performance. These findings strengthen the proposition of this paper that job insecurity undermines task performance acting as a hindrance stressor. As stated by Cavanaugh et al. (1998, p. 8), hindrance stressor refers “to work related demands or circumstances that tend to constrain or interfere with an individual’s work achievement, and that do not tend to be associated with potential gains for the individual.” In other words, job insecurity causes strain reactions (Lepine et al., 2005; Piccoli et al., 2021) and one way to emotionally cope with such a stressor is to behaviorally withdraw from the situation (Staufenbiel and König, 2010). Reduced task performance serves as a perfect example of such behavioral withdrawal (Staufenbiel and König, 2010).

Thus, based on theoretical reasoning and prevailing findings from primary studies and meta-analyses, the current paper hypothesizes the following:

**H6:** Job insecurity is directly and negatively related to task performance.

## Methodology

### Method

Partial least squares (PLS), a technique of structural equation modeling (SEM), can provide much value for causal inquiry in the Organisational Psychology field (Ringle et al., 2020). Following the procedure of Hair et al. (2019), this paper will report the results of an empirical study using PLS-SEM to validate a reflective structural model derived from the theoretical model previously developed here.

The PLS-SEM method will enable to estimate the model, with many constructs, indicators, and structural paths, without imposing the distributional normality on the data. In addition, PLS-SEM is considered a causal-predictive method (Sarstedt et al., 2017) that is very suitable for our purposes.

### Sample and Data Collection

Given the objective of the research, data were collected by using a convenience sampling type from employees in Lithuania. Convenience sampling is a type of non-probability sampling where members of the target population that meet certain practical criteria, such as availability at a given time, easy accessibility, geographical proximity, or the willingness to participate are included for the purpose of the study (Etikan et al., 2016).

For the survey, the online questionnaire was created. The questionnaire was distributed *via* LinkedIn, Facebook, Twitter, and Instagram. Due to the way of questionnaire dissemination, it is impossible to estimate the number of persons the questionnaire was sent to and the response rate. While distributing the questionnaire, the information about the purpose of the survey and a link to a survey were enclosed. Data collection took place during the COVID-19 lockdown period, in April and May 2020 (approx. 1 month). Such length of the period for data selection was chosen due to several reasons. First, as the study has been developed in a context characterized by VUCA, the period of approximately 1 month seems adequate as in VUCA context, and changes are continuous. Second, during the mentioned period, rules regarding lockdown have not been modified. Third, as usually 80% of responses are collected within 7 days (SurveyMonkey INC.),^1^ there was no advantage in keeping the survey open for a longer period. At the end of the research, 211 questionnaires were collected. The profile of respondents is presented in Table 1. Turning to demographical characteristics of the respondents, 163 of them were women (77.3%). Only 55 respondents (26.1%) held a managerial position. One hundred and six respondents were born in 1981–2001, and 88 respondents were born in 1965–1980.

**Table 1:** The respondents’ profile.

Characteristics	Frequency (*n*)	Percentage (%)
**Gender**		
Female	163	77.3
Male	46	21.8
Other	2	0.9
**Year of birth**		
Born in 2002 and later	4	1.9
Born in 1981–2001	106	50.2
Born in 1965–1980	88	41.7
Born in 1946–1964	13	6.2
**Work experience within the current organization**		
Up to 1 year	32	15.2
From 1 to 3 years	59	28.0
From 3 to 5 years	28	13.3
From 5 to 10 years	25	11.8
From 10 to 20 years	45	21.3
More than 20 years	22	10.4
**Position within the current organization**		
Managerial	55	26.1
Non-managerial	156	73.9

### Instrument

A self-reported questionnaire with questions to be answered on a five-point Likert scale was used in the study where 1 indicated “strongly disagree,” and 5 indicated “strongly agree.” All items were translated into Lithuanian language using a back translation procedure (Brislin, 1970), ensuring translation accuracy.

### Measures

Job insecurity was measured as a higher-order construct, which consists of two dimensions. First, quantitative job insecurity was measured using a four-item scale developed by De Witte (2000). Sample items are: “I feel insecure about the future of my job” and “I am sure I can keep my job” (reverse-coded). Second, qualitative job insecurity was measured using a four-item scale developed by Hellgren et al. (1999). Sample item is: “My pay development in this organisation is promising” (reverse-coded).

Trust in the organization was measured using seven items provided by Robinson (1996). Sample items are: “In general, I believe my employer’s motives and intentions are good” and “My employer is open and up-front with me.” The model also examined the SWB by using a five-item scale of Diener et al. (1985). A sample item is: “In most ways my life is close to my ideal.” Finally, task performance was measured using a four-item scale developed by Verburg et al. (2018). Sample item is “I fulfill the responsibilities specified in job description.”

## Results

The first step to assess the measurement model consists of examining the indicator loadings for verifying item reliability. Loadings above 0.7 are recommended, as they indicate that the construct explains more than 50 per cent of the indicator’s variance. Only two rounds were necessary to depurate indicators, one from trust in the organization and other from SWB.

The second step deals with assessing internal consistency reliability. In the model, composite reliability values are between 0.70 and 0.90, indicating from satisfactory to good, and any value is higher than 0.95, indicating that any indicator is redundant (Diamantopoulos and Winklhofer, 2001). Cronbach’s alpha, that is the classical measure of internal consistency reliability, and the alternative *rho*_A_ (Dijkstra and Henseler, 2015), that assumes similar thresholds, have also been calculated.

The third step consists of calculating the convergent validity of each construct measure. The metric used is the average variance extracted (AVE) that has to be 0.50 or higher. That occurs in our model, indicating that the constructs explain more than 50 per cent of the variance of its respective indicators.

Table 2 shows the results from the measurement model commented above in detail.

**Table 2:** Convergent validity and internal consistency reliability.

Construct	Item	Convergent validity		Internal consistency reliability		
		Loadings >0.70	AVE >0.50	Composite reliability >0.70	Reliability (*rho*_A_) >0.70	Cronbach’s Alfa 0.65–0.95
JI	QUAN	0.902	0.786	0.880	0.738	0.729
	QUAL	0.871				
TO	TO1	0.870				
	TO2	0.836	0.662	0.921	0.905	0.897
	TO4	0.847				
	TO5	0.714				
	TO6	0.826				
	TO7	0.781				
SWB	SWB1	0.764	0.656	0.884	0.835	0.824
	SWB2	0.870				
	SWB3	0.855				
	SWB4	0.744				
EP	EP1	0.739	0.600	0.857	0.781	0.777
	EP2	0.813				
	EP3	0.811				
	EP4	0.733				

The fourth step is to assess the extent to which the constructs in the model are empirically distinct one from other, i.e., to check the discriminant validity of each construct. For that purpose, we have calculated both, the classical Fornell–Larcker measure and the HTMT, the newest ratio developed by Henseler et al. (2015), that must be above 0.90 (Table 3).

**Table 3:** Discriminant validity.

Constructs	Fornell–Larcker criterion				Heterotrait–Monotrait ratio			
	JI	TO	SWB	EP	JI	TO	SWB	OT
JI	0.88							
TO	−0.60	0.81			0.74			
SWB	−0.46	0.49	0.81		0.58	0.56		
EP	−0.30	0.37	0.43	0.77	0.40	0.43	0.53	

Once the measurement model has been assessed, the fifth step is to assess the structural model. Before, we have verified that the model has no collinearity problems by checking the VIF value (Hair et al., 2019). All VIF values were close to 3 and above, ranging from the lower 1.44 from the indicator EP4, to the highest 2,940 from the indicator TO1.

For the purpose of assessing the structural model, we have first calculated the coefficient of determination (*R*^2^). Second, we have calculated the blindfolding-based cross-validated redundancy measure *Q*^2^. The *R*^2^ for the three endogenous constructs is acceptable indicating good model’s explanatory power. In the same way, the *Q*^2^ value. As a rule of thumb, *Q*^2^ values are higher than 0, showing the predictive relevance of the model. These results are shown in Table 4.

**Table 4:** Assessment criteria *R*^2^ and *Q*^2^ for the structural model.

Constructs	*R* ^2^	*Q* ^2^
TO	0.363	0.235
SWB	0.291	0.185
EP	0.201	0.112

Third, and finally, we have calculated the statistical significance and relevance of the path coefficients through the bootstrapping procedure, with 5,000 resamples (Table 5).

**Table 5:** Hypotheses testing.

Hypothesis	Path coefficient (original)	Path coefficient (sample)	St. Error	Confidence interval [2.5/97.5]%	t-statistics	Significant (*p* < 0.05; accepted or rejected)
**H2:** TO SWB	0.340	0.345	0.088	0.151/0.500	3.863	0.000 (accepted)
**H3:** SWB EP	0.369	0.375	0.074	0.214/0.502	4.974	0.000 (accepted)
**H4:** JI TO	−0.602	−0.606	0.045	−0.681/−0.502	13.348	0.000 (accepted)
**H5:** JI SWB	−0.262	−0.260	0.097	−0.447/−0.063	2.696	0.007 (accepted)
**H6:** JI EP	−0.136	−0.137	0.075	−0.275/0.016	1.821	0.069 (rejected)

## Discussion

The paper was intended to examine the relationship between job insecurity, trust in the organization, SWB, and task performance. More specifically, treating job insecurity as a hindrance stressor, the paper claims for a negative association between job insecurity and the mentioned outcomes. In doing this, the paper echoes the call in the previous literature to focus on employee well-being, attitudinal outcomes, and performance as the three major outcomes of job insecurity (Lee et al., 2018; Shin et al., 2019) acknowledging the VUCA environment. Further, the paper addresses the relationship between constructs in the virtuous cycle. More specifically, the paper analyzes how trust in the organization, SWB, and task performance are related. Turning to the methodological part, the reflective measurement model tested has provided acceptable item reliability that has been verified in all constructs, including job insecurity, confirming its dimensions. Consequently, H1 has been verified. The convergent and the discriminant validity of all constructs in the model have also been verified. Turning to the structural model, the statistical significance and relevance of the path coefficients have been verified for H2, H3, H4, and H5, with H6 as the only rejected hypothesis. Further, the theoretical and practical implications of the findings are discussed.

### Theoretical Implications

First, the previous literature distinguishes between considered quantitative and qualitative job insecurity (Lee et al., 2018). However, the paper provides support that job insecurity is multidimensional construct, confirming its two dimensions.

Second, as job insecurity is a subjective experience (Vander Elst et al., 2014a), employees may experience varying degrees of uncertainty, even if they are objectively under the same working conditions (Lepine et al., 2005). As such, job insecurity may trigger contradicting reactions. This notwithstanding, the growing body of the literature considers job insecurity as a relevant job stressor, which has a detrimental effect on employees (Lee et al., 2018).

As it was predicted, the findings revealed that job insecurity served as a determinant of lower trust in the organization. These findings are in line with some previous studies (Kim, 2019) supporting the idea that breach of the psychological contract harms the employer–employee relationships (Maguire, 2003; Rao, 2021). As trust creates a collaborative environment by giving employee a feeling of security (Dirks and Ferrin, 2001; Ertürk and Vurgun, 2015), it is particularly important in times of crisis and uncertainty (Gustafsson et al., 2021), like in the VUCA world. Hence, the current paper contributes by elaborating on the relationship between trust in the organization and overall employee concern about the continued existence of their job in the future and its valued features (De Witte, 2005).

Third, as predicted, the findings revealed that job insecurity led to lower SWB. According to the COR theory, when employees perceive a resource loss or anticipate the possibility of resource loss (perception of job insecurity), they will invest their remaining resources in proactive defense against such resource loss (Hobfoll, 1989). As such, the defense against the potential loss of job (quantitative job insecurity) or valued job features (qualitative job insecurity) might result in lower SWB. Although some previous studies have confirmed the hypothesized negative effect of job insecurity on SWB, mostly they considered quantitative job insecurity or qualitative job insecurity (Hu et al., 2021). Hence, the current paper broadens the literature treating job insecurity as a second-order construct and providing an answer to the empirical question regarding the potential negative impact of job insecurity on SWB.

Fourth, contrary to the expectations, the hypothesis regarding the negative effect of job insecurity on task performance was rejected. Previous findings were contradicted. Some studies have shown that job insecurity decreased task performance of employees (Cheng and Chan, 2008; Schreurs et al., 2012). However, other studies have shown that job insecurity was not related to performance or even have suggested that job insecurity could motivate employees to perform better in order to secure their jobs (Shin et al., 2019; Sverke et al., 2019). Accordingly, the current finding calls for further investigation and stimulates further discussion through the understanding of the relationship between job insecurity and task performance as job insecurity might serve in this relationship as a hindrance stressor or challenge stressor (Piccoli et al., 2021).

Fifth, while exploring the virtuous cycle between trust in the organization, SWB, and task performance, the findings demonstrated that trust in the organization increased SWB, whereas SWB increased task performance. Hence, the current paper broadens the literature by exploring the virtual cycle between constructs, which are important outcomes of job insecurity.

### Practical Implications

In addition to the theoretical implications, the research has some managerial implications for practitioners. Following the notion that job insecurity is one of the most important stressors in work life (Vander Elst et al., 2014a) and based on the finding that job insecurity impairs trust in the organization and task performance, organizational leaders are invited to design some strategies and take some initiatives, which are concerned with eliminating or reducing job insecurity as such. The current literature supports the initial view of De Witte (2005) that job insecurity could be eliminated or mitigated by communication (Jiang and Probst, 2014), participation in decision making (Gallie et al., 2017), and enhancement of organizational justice (Greenberg, 1990). More recently, Shin and Hur (2019) highlighted the importance of employee training aimed at increasing their confidence. Although job insecurity is inevitable in the VUCA world, several aspects that might be taken into consideration by practitioners are provided below.

First, valuable and relevant communication might serve as an energy resource (Jiang and Probst, 2014). Accordingly, open, early, and honest information increases the predictability of future work existence and its valued features (De Witte, 2005). Moreover, communication tends to show that one is respected as an employee (De Witte, 2005). Respect captures the state of being seen and valued by recognizing another person, listening, understanding, and appreciating people, attending to needs, emphasizing another’s good qualities (Carmeli et al., 2015). Thus, leaders are strongly encouraged to implement a sustainable communication process where frequency, channels, structure, and content of the messages are highly important.

Second, by participating, employees have influence over decision making (Vander Elst et al., 2010) and control over situation (De Witte, 2005); therefore, job insecurity is reduced. Consulting with employees on work-related issues spreads the message among employees that their needs are important for the organization and taken into consideration (Gallie et al., 2017). Clear strategy addressing employee participation in decision making and implementation of explicit actions lead to higher situation predictability and control, which in turn mitigate job insecurity.

Third, the organizations should rethink organizational justice, which deals with the understanding of the complexity of fair treatment in a work setting (Graso et al., 2020). In fact, employees who perceive greater organizational justice will have a stronger sense of being valued by the organizations (Cropanzano et al., 2001). The sense of value tends to increase the predictability and controllability of work situations experienced by employees and accordingly lowers their job insecurity.

Fourth, the role of employee development, especially in a VUCA world (Dachner et al., 2021), is highly underestimated in the literature. The employee development referring to “the expansion of an individual’s capacity to function effectively in his or her present or future job and work organization” (McCauley and Hezlett, 2001, p. 314) is supposed not only to enhance the employee competences, but also reduce job insecurity, as employee will be more confident about successful managing of changes in case of job or its valued attribute loss.

Summing up, the complex of actions with respect to open communication, employee development, involving employees in decision making, and increasing the feeling of organizational justice of the organizational actions might create a synergic effect and reduce job insecurity as such.

### Limitations

This research has some shortcomings that might be addressed in future research.

The first concern is related to self-reported nature of the data regarding task performance. This may have increased the risk of common method variance (Podsakoff et al., 2003, 2012) and other response biases such as social desirability. Attempts were made to decrease the social desirability on measurements of task performance by guaranteeing the anonymity of results and emphasizing that there would be no right or wrong answers (Piccoli et al., 2021). Nonetheless, in order to avoid overrated results, other rated measures of task performance are recommended (Peiró et al., 2019).

The second concern deals with the fact that objective predictors of job insecurity were neglected in the current research. In the course of the survey, the data on organizations’ industry or size, or employee income level, educational level or living place (rural or urban area) were not collected. Assuming that objective predictors matter (Keim et al., 2014), further research should consider previously mentioned relevant data.

The third concern is related to the sample size. The sample size limits the opportunity to draw generalized conclusions. The paper calls for the future studies addressing the sample which would allow for providing robust generalized conclusions.

The fourth concern refers to the sample. As a sample from one country is considered to be an appropriate practice (Shin and Hur, 2019), it would nonetheless be interesting to examine whether the job insecurity, trust in the organization, SWB, and task performance relationship in a virtuous cycle vary across countries and whether this variation depends on specific country-level characteristics.

Finally, seeing that the previous studies showed inconsistent results regarding the existing gender differences when predicting the perceived job insecurity based on objective individual and organizational variables (Menéndez-Espina et al., 2020), further studies might address the gender aspect while revealing the linkage between job insecurity, trust in the organization, SWB, and task performance in the VUCA context.

## Conclusion

The aim of the paper was to explore the linkage between job insecurity, trust in the organization, SWB, and task performance in the VUCA context while addressing the virtuous cycle. The findings confirmed that job insecurity could be treated as a bidimensional construct capturing both qualitative and quantitative dimensions. Further, the results revealed that job insecurity reduced employee trust in the organization and their SWB, while the hypothesis regarding the detrimental impact of job insecurity on task performance was rejected. Moreover, findings in the virtuous cycle allowed for concluding that employees who trusted their organizations more felt happier and accordingly happier employees performed better while dealing with job responsibilities included in the job description. Treating job insecurity as a stressor and seeing that job insecurity is inevitable in the contemporary VUCA world, organizations are encouraged to deal with unpredictability and uncontrollability of work situations experienced by employees and thus reduce job insecurity.

## Data Availability Statement

The raw data supporting the conclusions of this article will be made available by the authors, without undue reservation.

## Author Contributions

ŽS, M-SH, and ES conceived and designed the work, and drafted the article. ŽS and ES collected the data. M-SH analyzed and interpreted the data. ŽS critically revised the article and approved the published version. All authors contributed to the article and approved the submitted version.

## Conflict of Interest

The authors declare that the research was conducted in the absence of any commercial or financial relationships that could be construed as a potential conflict of interest.

## Publisher’s Note

All claims expressed in this article are solely those of the authors and do not necessarily represent those of their affiliated organizations, or those of the publisher, the editors and the reviewers. Any product that may be evaluated in this article, or claim that may be made by its manufacturer, is not guaranteed or endorsed by the publisher.

## Footnotes

^1^ www.surveymonkey.com
